# Three-minute bench step exercise as a countermeasure for acute mental stress-induced arterial stiffening

**DOI:** 10.1371/journal.pone.0279761

**Published:** 2022-12-30

**Authors:** Daisuke Kume, Masato Nishiwaki, Ryo Takahara, Norio Hotta

**Affiliations:** 1 Faculty of Information Science and Technology, Osaka Institute of Technology, Hirakata, Osaka, Japan; 2 Faculty of Engineering, Osaka Institute of Technology, Asahi-ku, Osaka, Japan; 3 Tatami Incorporated, Nakahara-ku, Kawasaki, Japan; 4 Graduate School of Engineering, Chiba University, Inage-ku, Chiba, Japan; 5 Department of Lifelong Sports and Health Sciences, Chubu University, Kasugai, Aichi, Japan; University of Virginia, UNITED STATES

## Abstract

Acute mental stress (MS) induces a transient increase in arterial stiffness. We verified whether a single bout of bench step (BS) exercise for 3 minutes counteracts acute MS-induced arterial stiffening. Fifteen healthy young men (mean age, 21.7 ± 0.3 years) underwent two experimental trials: rest (RE) and exercise (EX) trials. Following a 5-minute MS task, the participants in the RE trial rested on a chair for 3 minutes (from 10 to 13 minutes after task cessation), whereas those in the EX trial performed the BS exercise for the same duration. The heart-brachial pulse wave velocity (PWV) (hbPWV), brachial-ankle PWV (baPWV), heart-ankle PWV (haPWV), and the cardio-ankle vascular index (CAVI) were measured at baseline and at 5 and 30 minutes after the task. In both trials, significant increases in hbPWV, haPWV, and CAVI occurred at 5 minutes after the task; these elevations persisted until 30 minutes after the task in the RE trial, but significantly decreased to baseline levels in the EX trial. baPWV was significantly elevated at 30 minutes after the task in the RE trial, but not in the EX trial. This study reveals that a 3-minute BS exercise offsets acute MS-induced arterial stiffening.

## Introduction

Arterial stiffening is an independent risk factor for future cardiovascular disease [1], and pulse wave velocity (PWV) is an established index of arterial stiffness. It is generally understood that arterial stiffness increases with advancing age, obesity, diabetes, and dyslipidemia [2, 3], whereas it can be decreased by appropriate therapeutic interventions, such as regular exercise training [4].

Chronic mental stress (MS) is also known to be detrimental for vascular health [5]. Importantly, even a brief acute episode of MS induces a transient increase in arterial stiffness as assessed by the PWV [6–10]. Both acute response and chronic adaptation of the vasculature to MS have been indicated to be probably relevant [11, 12]; thus, repeated exposures to episodic increases in arterial stiffness and its accumulation due to acute MS in daily life may lead to an increase in the basal levels of arterial stiffness. Therefore, it is crucial to formulate a practical and effective strategy to counteract acute MS-induced arterial stiffening.

Our group previously reported that performing a 10-minute cycling exercise immediately after acute MS can counteract stress-induced arterial stiffening [7]. In a subsequent study [8], to improve the applicability of this exercise strategy in real life, we used a bench step (BS) exercise that can be conveniently performed in different settings, including the office and home. The results indicated that 10-minute BS exercise after acute MS was effective in offsetting stress-induced arterial stiffening; however, the exercise had only a limited effect against subsequent acute MS. Our findings may provide a basis to recommend engaging in brief exercise every time a stressful situation arises. Therefore, feasible short-duration exercises are warranted, as time requirement is one of the most common barriers to exercise. In this regard, previous studies identified potential health benefits derived from rapid bouts (i.e., within 5 minutes) of exercise. For instance, some studies reported that 5-minute exercise could transiently decrease arterial stiffness measures [13–15]. Additionally, exercise for even 3 minutes has been reported to ameliorate postprandial hyperglycemia [16]. Furthermore, interrupting prolonged sitting with 3-minute bouts of exercise lowers resting blood pressure (BP) and sympathetic activity [17]. Based on these findings, even a brief bout of BS exercise may be a countermeasure for acute MS-induced arterial stiffening. In this study, we tested the hypothesis that a single bout of BE exercise for as short as 3 minutes could counteract acute MS-induced arterial stiffening.

## Methods

### Participants

This study employed a randomized crossover trial study design. The sample size required for this study was calculated using G*Power version 3.1 (Heinrich Heine University Düsseldorf, Düsseldorf, Germany). Based on our previous studies [6–8], the effect size (ES) was set to 0.25 using a within-between interaction of two-way repeated-measures analysis of variance (ANOVA). The α- and β-levels were set to 0.05 and 0.2 (80% power), respectively. The minimum number of participants required was determined to be 12.

Fifteen healthy young men recruited from the Osaka Institute of Technology were included as study participants (means ± SE; age, 21.7 ± 0.3 years; height, 169.8 ± 1.2 cm; body weight, 61.0 ± 1.6 kg; and body mass index, 21.2 ± 0.5 kg/m^2^). Recruitment was conducted through local advertisements and referrals. None of the participants were smokers or were receiving any medication. All participants provided written informed consent, and the study purpose, experimental procedure, and associated risks were fully explained to them. The study was approved by the Human Ethics Committee of the Osaka Institute of Technology (approval number: #2021–102) and was conducted in accordance with the guidelines of the Declaration of Helsinki.

### Experimental procedures

The participants visited the laboratory three times throughout the experimental period. During the first visit, the participants were familiarized with the experimental apparatus. During the second and third visits (i.e., experimental visits), the participants were subjected to the rest (RE) and exercise (EX) trials. The order of experimental visits was randomly allocated, and consecutive visits were separated by an interval of approximately 1 week. All experimental sessions were conducted in a quiet air-conditioned room (24–26°C). For each participant, the experiments were conducted at the same time of the day in order to avoid any potential diurnal effects. The participants were asked to refrain from performing strenuous exercise, consuming alcohol (≥24 h), caffeine (≥12 h), or eating (≥3 h) before trials.

**Fig 1:**
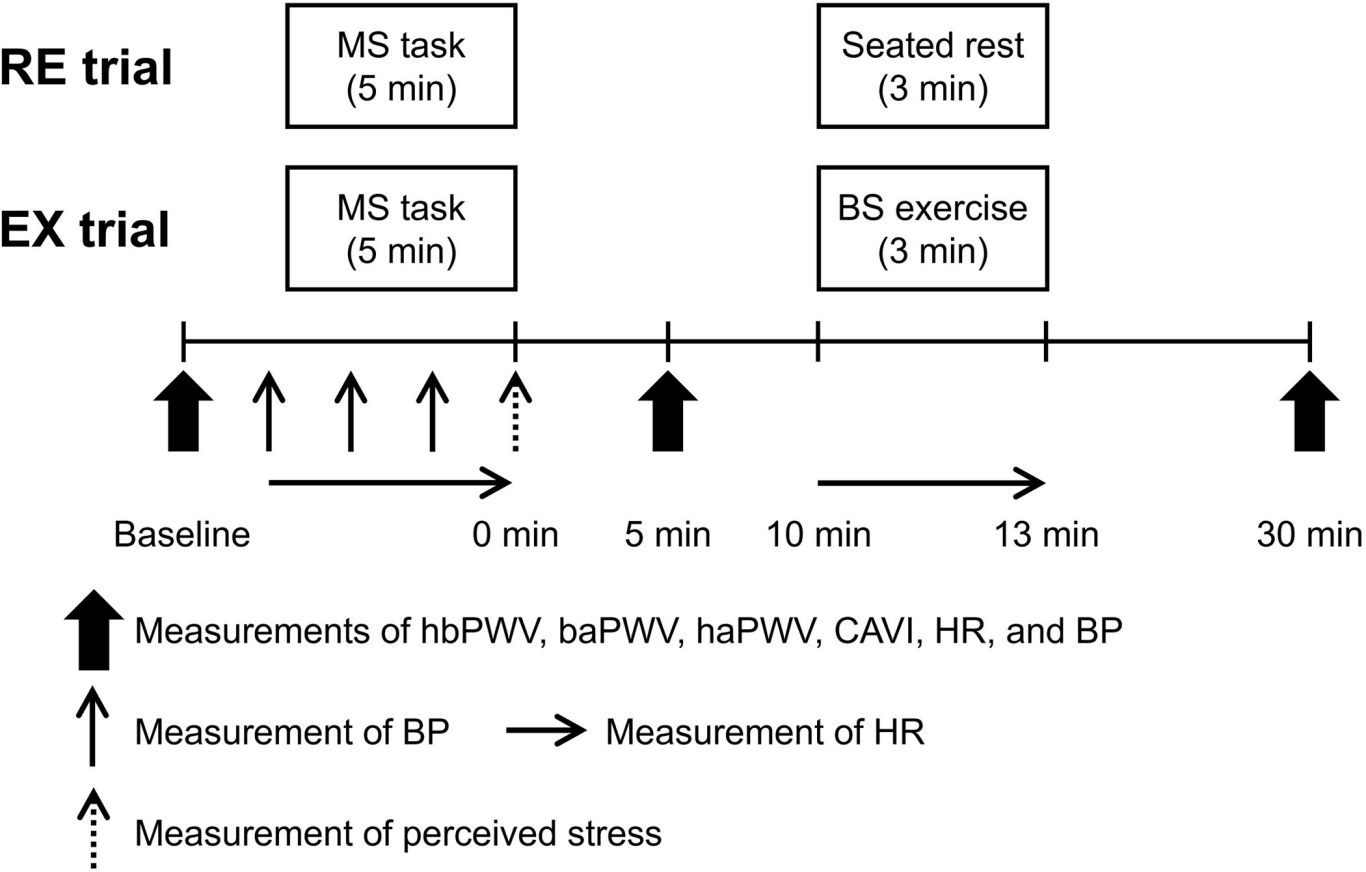
Experimental protocol. RE, rest; EX, exercise; MS, mental stress; BS, bench step; hbPWV, heart-brachial pulse wave velocity; baPWV, brachial-ankle pulse wave velocity; haPWV, heart-ankle pulse wave velocity; CAVI, cardio-ankle vascular index; HR, heart rate; BP, blood pressure.

A schematic representation of the experimental protocol of this study is presented in Fig 1. In both trials, baseline measurements were obtained with the participants in the supine position after a 15-minute rest period. The participants then performed a 5-minute MS task, and the post-task phase was set to 30 minutes. Subsequently, the participants in the RE trial rested on a comfortable chair for 3 minutes (from 10 to 13 minutes after the completion of the task), whereas those in the EX trial performed the BS exercise for 3 minutes using a step bench (Stepwell 2; Konami, Tokyo, Japan) set at a height of 20 cm. The step rhythm was individualized for each participant to target a rate of perceived exertion (RPE) of 11–13 (Borg scale, 6–20) because it was difficult to regulate exercise intensity with steady-state heart rate (HR) values as in our previous studies [7, 8] due to the short exercise duration; the target RPE was set according to a previous report [18]. Except for the seated rest or exercise periods, the participants remained in the supine position throughout the experimental session.

Based on our previous studies [6–8], we used mental arithmetic as the MS task. The participants were asked to serially subtract 13 from a 3-digit number as quickly and accurately as possible for a period of 5 minutes. During the task, the participants were instructed to perform the task faster and were immediately corrected if wrong answers were provided in order to intentionally cause frustration. A metronome was played loudly for additional distraction. When the number was <13 (the answer was not allowed to go below 0), the participants restarted the task using the original 3-digit number.

### Measurements

In addition to the aforementioned baseline measures, PWV, HR, systolic and diastolic BP (SBP and DBP), and mean arterial pressure (MAP) were measured at 5 and 30 minutes after the MS task using a vascular testing system (VaSera VS-1500AN; Fukuda Denshi, Tokyo, Japan); the last measurement timepoint (e.g., 30 minutes after the task) was set because impairment of vascular function at 30 minutes after stress exposure was reported to be associated with future cardiovascular events [12]. For these measurements, BP cuffs were wrapped around both the upper arms and ankles. Electrocardiograph (ECG) electrodes and a phonocardiograph microphone were placed on both wrists and on the pectoral region, respectively. ECG, heart sounds, and arterial pressure waveforms at the brachial and posterior-tibial arteries were simultaneously recorded. According to our previous studies [6–8], we measured heart-brachial PWV (hbPWV), brachial-ankle PWV (baPWV), and heart-ankle PWV (haPWV). Specifically, hbPWV, baPWV, and haPWV were calculated from each arterial path length along with the time intervals between the second heart sound and the dicrotic notch on the brachial arterial pressure waveform, between the foot of the brachial arterial pressure waveform and the foot of the posterior-tibial arterial waveform, and the sum of these time intervals [19–21]. Moreover, the cardio-ankle vascular index (CAVI) was automatically calculated. hbPWV reflects the stiffness from the heart to the brachial artery and can serve as a marker of proximal aortic stiffness [20], baPWV reflects the stiffness of the abdominal aorta and leg arteries, haPWV reflects the stiffness from the aorta to the ankle, and CAVI is a parameter of haPWV adjusted by BP [22]. In our laboratory, the day-to-day coefficients of variation for hbPWV, baPWV, haPWV, and CAVI were 2.4 ± 0.2%, 1.5 ± 0.1%, 1.5 ± 0.1%, and 2.5 ± 0.3%, respectively.

Before and during the MS task, SBP, DBP, and MAP were measured using an automated sphygmomanometer (Tango M2; SunTech Medical Instruments, North Carolina, USA). BP was measured twice at approximately 2 and 4 minutes after the beginning of the task, and the average value was calculated. HR was also measured continuously using a three-lead ECG (413; Intercross, Tokyo, Japan). The HR data obtained concurrently with the BP measurements were averaged. Furthermore, after completing each task, the participants were asked to rate their perceived stress during the task using a standard five-point scale of 0 (not stressful), 1 (somewhat stressful), 2 (stressful), 3 (very stressful), and 4 (very, very stressful) [23].

HR during the BS exercise was continuously recorded using a wireless HR monitor (m430; Polar Electro Oy, Kempele, Finland). Additionally, HR was obtained during seated rest. The HR data during the last 60 s of each trial were averaged. In the EX trial, the number of steps was counted, and the step rhythm per minute was calculated. Additionally, immediately after cessation of the exercise, the participants reported the RPE that was actually felt during the exercise.

### Statistical analysis

Data are presented as means ± SE. The perceived stress levels pertaining to the MS task were compared between the trials using a paired Student’s *t* test. Two-way (time × trial) repeated-measures analysis of variance (ANOVA) with Bonferroni-corrected post-hoc testing was performed for the measures of arterial stiffness and hemodynamic variables.

Statistical significance was considered at a *P-*value of <0.05. All analyses were performe using SPSS version 28.0 (IBM SPSS Japan, Tokyo, Japan). With respect to the arterial stiffness measures, ES (dz) was calculated between the values of interest using G*Power version 3.1 [24, 25].

## Results

In the RE trial, the average HR value during seated rest was 71 ± 2 bpm. In the EX trial, the HR value during exercise was 133 ± 2 bpm, which corresponded to 49.1 ± 2.1% of HR reserve (HRR); the step rhythm and RPE were 131 ± 3 steps/minute and 12.1 ± 0.1, respectively.

HR and BP measures significantly increased in response to the MS task in both trials, with no significant differences between the trials (Table 1). There was also no significant difference in the perceived stress level between the trials (RE vs. EX trials, 2.7 ± 0.3 vs. 2.9 ± 0.2).

**Table 1:** Hemodynamic variables before and during the MS task.

	Trial	Before the task	During the task	ANOVA
HR (bpm)	RE	63 ± 2	79 ± 3*	Time: *F* = 92.151, *P* < 0.001; Trial: *F* = 2.678, *P* = 0.124
	EX	66 ± 2	81 ± 2*	Interaction: *F* = 0.077, *P* = 0.785
SBP (mmHg)	RE	115 ± 2	131 ± 3*	Time: *F* = 101.383, *P* < 0.001; Trial: *F* = 1.416, *P* = 0.254
	EX	116 ± 2	133 ± 3*	Interaction: *F* = 0.501, *P* = 0.491
DBP (mmHg)	RE	65 ± 1	82 ± 2*	Time: *F* = 50.126, *P* < 0.001; Trial: *F* = 0.092, *P* = 0.766
	EX	67 ± 1	81 ± 2*	Interaction: *F* = 0.900, *P* = 0.359
MAP (mmHg)	RE	82 ± 1	98 ± 2*	Time: *F* = 66.027, *P* < 0.001; Trial: *F* = 1.281, *P* = 0.277
	EX	83 ± 1	98 ± 2*	Interaction: *F* = 0.255, *P* = 0.621

Data are expressed as means ± SE

HR, heart rate; SBP, systolic blood pressure; DBP, diastolic blood pressure; MAP, mean arterial pressure; RE, rest; EX, exercise; ANOVA, analysis of variance

*P < 0.05 vs. before the task

**Fig 2:**
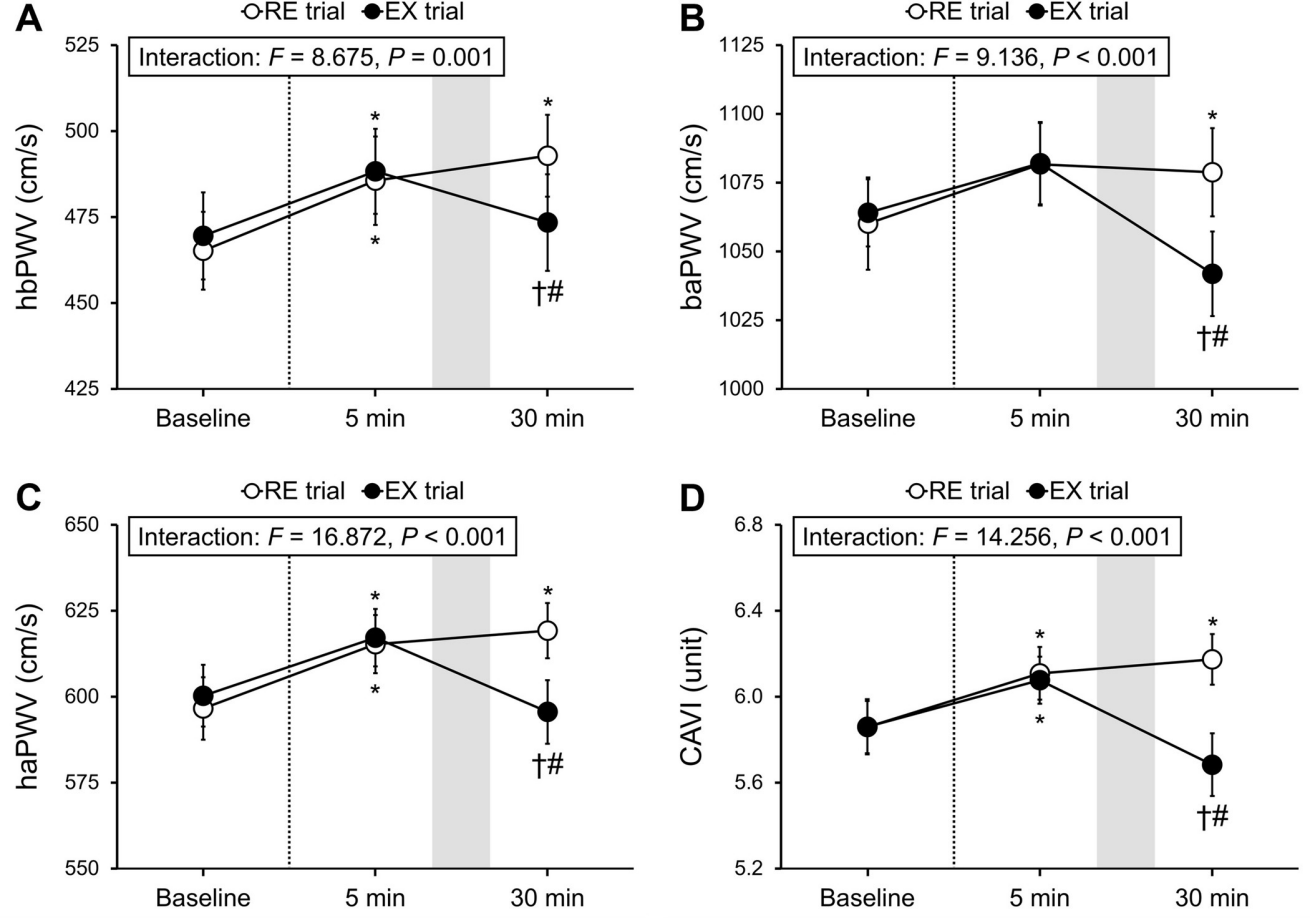
Measurements of hbPWV (**a**), baPWV (**b**), haPWV (**c**), and CAVI (**d**). The dashed lines indicate the time point of the MS task. The shaded boxes indicate the duration of seated rest or bench step exercise. RE, rest; EX, exercise; hbPWV, heart-brachial pulse wave velocity; baPWV, brachial-ankle pulse wave velocity; haPWV, heart-ankle pulse wave velocity; CAVI, cardio-ankle vascular index; MS, mental stress. *P < 0.05 vs. baseline. †*P* < 0.05 vs. 5 min after the task. #*P* < 0.05 vs. the RE trial. Data are expressed as means ± SE.

The results of arterial stiffness measurements are presented in Fig 2. In both trials, hbPWV (ES = 1.13 and 1.38 for the RE and EX trials, respectively), haPWV (ES = 1.40 and 1.71 for the RE and EX trials, respectively), and CAVI (ES = 1.07 and 1.00 for the RE and EX trials, respectively) significantly increased at 5 minutes after the MS task. The values remained elevated at 30 minutes after the task in the RE trial, but significantly decreased to baseline levels in the EX trial (ES = 0.81, 1.28, and 1.13 for hbPWV, haPWV, and CAVI, respectively). Additionally, in both trials, baPWV increased at 5 minutes after the task (ES = 0.74 and 0.65 for the RE and EX trials), although this did not reach statistical significance. Subsequently, a significant increase in baPWV occurred at 30 minutes after the task in the RE trial; however, it significantly decreased to baseline levels in the EX trial (ES = 1.13). All arterial stiffness measures were significantly lower in the EX trial than in the RE trial at 30 minutes after the task (ES = 0.75, 0.96, 1.21, and 1.27 for hbPWV, baPWV, haPWV, and CAVI, respectively).

Changes in hemodynamic variables are summarized in Table 2. HR and SBP increased 5 minutes after the MS task in both trials, but there was only statistical significance in the EX trial. Thirty minutes after the task, HR was significantly higher in the EX trial than in the RE trial; MAP was also significantly elevated in the EX trial.

**Table 2:** Time-course changes in hemodynamic variables.

	Trial	Baseline	5 min	30 min	ANOVA
HR (bpm)	RE	64 ± 2	66 ± 2	62 ± 2	Time: *F* = 9.611, *P* < 0.001; Trial: *F* = 13.820, *P* = 0.002
	EX	65 ± 1	68 ± 1*	68 ± 1*#	Interaction: *F* = 4.001, *P* = 0.030
SBP (mmHg)	RE	117 ± 2	119 ± 2	118 ± 1	Time: *F* = 4.141, *P* = 0.027; Trial: *F* = 7.750, *P* = 0.015
	EX	118 ± 1	121 ± 1*	120 ± 2#	Interaction: *F* = 1.183, *P* = 0.321
DBP (mmHg)	RE	71 ± 1	73 ± 2	73 ± 1	Time: *F* = 4.249, *P* = 0.024; Trial: *F* = 0.825, *P* = 0.379
	EX	72 ± 4	73 ± 2	74 ± 1	Interaction: *F* = 0.728, *P* = 0.492
MAP (mmHg)	RE	86 ± 1	88 ± 2	88 ± 1	Time: *F* = 5.297, *P* = 0.011; Trial: *F* = 2.956, *P* = 0.108
	EX	87 ± 1	89 ± 1	90 ± 1*	Interaction: *F* = 1.372, *P* = 0.270

Data are expressed as means ± SE

HR, heart rate; SBP, systolic blood pressure; DBP, diastolic blood pressure; MAP, mean arterial pressure; RE, rest; EX, exercise; ANOVA, analysis of variance

*P < 0.05 vs. baseline

#*P* < 0.05 vs. the RE trial

## Discussion

In this study we found that elevations in hbPWV, baPWV, haPWV, and CAVI owing to acute MS were completely abolished by performing a 3-minute BS exercise thereafter (i.e., EX trial), whereas these elevations remained sustained in the RE trial. The data from the present study support our hypothesis and provide novel practical evidence that a BS exercise for only 3 minutes is sufficiently effective in offsetting acute MS-induced arterial stiffening.

Both trials in our study showed an elevation in arterial stiffness measures at 5 minutes after the MS task; however, the increase in baPWV did not reach statistical significance and remained elevated for 30 minutes after the task when the participants rested, as in the RE trial. The current observations are supported by the findings of numerous studies, which reported an increase in arterial stiffness following acute MS [6–10, 26, 27]. It is considered that the detrimental vascular effects induced by acute MS are attributable to various physiological factors, including changes in the endothelial function, sympathetic nerve activity, hormonal status, inflammation, and oxidative stress [6–8, 10, 27, 28].

The most important finding of this study is that the 3-minute BS exercise completely abolished the elevations in hbPWV, baPWV, haPWV, and CAVI in the EX trial. This finding builds upon our prior work by demonstrating that even a mere 3-minute BS exercise can counteract acute MS-induced arterial stiffening. Although largely speculative, we believe that the following are the physiological mechanisms underlying this effect. The synthesis of nitric oxide, an endothelial-derived vasodilatory factor, is augmented even from short-term exercises [29]; indeed, a 10-minute bout of exercises has been reported by some studies to improve the endothelial function, which is an important determinant of arterial stiffness [18, 30]. Moreover, metabolites derived from exercised muscles reportedly attenuate arterial smooth muscle tone and consequently reduce arterial stiffness [14]. Therefore, we speculate that exercise-induced changes in the endothelial function and the release of muscle metabolites might counteract the mechanisms underlying arterial stiffening caused by acute MS. However, it is important to note that the previous studies used to support our speculation involved exercises that were performed over a longer duration (i.e., 5–10 minutes) than the exercises in this study. Further studies are needed to clarify the relevant mechanisms.

In our previous studies [7, 8], which showed that 10-minute cycling or BS exercises following acute MS could offset the stress-induced arterial stiffening, the exercise intensities were 35.4% and 38.8% of the HRR, respectively, which is considered as light-intensity aerobic exercise [31]. As stated above, in this study, in which 3-minute exercise was performed, RPE was used to regulate the exercise intensity as HR values could not reach a steady-state. Consequently, the participants achieved 49.1% of HRR, which is classified as moderate-intensity exercise [31]. The minimum exercise intensity to offset the detrimental vascular effects of acute MS in this setting has not yet been elucidated. Clarifying this point would further improve the applicability of this exercise strategy in real life as it is considered that common barriers to exercise include not only time consumption, but also perceived exertion.

Owing to the COVID-19 pandemic, the number of individuals working from home has dramatically increased [32]. Currently, the number of employees working from home remains higher than that prior to the pandemic [33]. It is predicted that hybrid working, in which work is performed in both the office and home, will become more prevalent and established in the near future. Because a BS exercise can be readily performed at home as well as in the office, we recommend performing a BS exercise for 3 minutes every time a stressful situation arises as a practical and effective strategy for protecting the vasculature from daily stress.

One limitation of this study is that only healthy young men were included. Thus, it will be necessary in future studies to evaluate whether our findings can be extrapolated to other populations.

## Conclusion

This study demonstrates that performing BS exercise for only 3 minutes can counteract acute MS-induced arterial stiffening.

## Supporting information

S1 Dataset (XLSX)
